# Survey of otitis externa in American Cocker Spaniels in Finland

**DOI:** 10.1186/s13028-017-0282-3

**Published:** 2017-02-28

**Authors:** Mirja Kaimio, Leena Saijonmaa-Koulumies, Outi Laitinen-Vapaavuori

**Affiliations:** Department of Equine and Small Animal Medicine, Faculty of Veterinary Medicine, University of Helsinki, P.O. Box 57, 00014 Helsinki, Finland

**Keywords:** American Cocker Spaniel, Otitis externa, Prevalence, End-stage otitis externa, Owner questionnaire

## Abstract

**Background:**

American Cocker Spaniels are overrepresented among breeds that require surgery as a treatment of end-stage otitis externa. However, the prevalence of otitis externa (OE) in this breed remains unknown. We reviewed the year 2010 medical records of 55 private veterinary clinics in Finland to determine the prevalence of OE in American Cocker Spaniels compared with English Cocker and English and Welsh Springer Spaniels. An American Cocker Spaniel owner questionnaire was designed to identify potential risk factors for end-stage OE.

**Results:**

From the medical records of 98,736 dogs, the prevalence of OE was highest in Welsh Springer Spaniels (149 out of 468, 31.8%, [95% confidence interval 27.6–36.0]), followed by American Cocker (89/329, 27.0%, [22.2–31.7]), English Springer (96/491, 19.6%, [16.1–23.1]) and English Cocker Spaniels (231/1467, 15.7%, [13.8–17.6]). The mean number of OE episodes in ear-diseased dogs and the number of ear surgeries were highest in American Cocker Spaniels. Owner questionnaires were received for 151 American Cocker Spaniels, 85 (56%) of which had suffered from OE. In 47% (40/85) of these dogs, OE occurred without concurrent skin lesions, 46% (33/72) displayed the first signs of OE before 1 year of age. In 24% (20/85) of the dogs, the signs of OE recurred within 1 month or continued despite treatment, 16% (14/85) required surgery (n = 11) or were euthanized (n = 5; 2 of the operated dogs and 3 others) due to severe OE. The onset of OE before the age of 1 year significantly increased the risk (OR 3.8, 95% CI 1.1–13.6) of end-stage OE.

**Conclusions:**

The prevalence of OE in American Cocker Spaniels in Finland was higher than previously reported in Cocker Spaniels, but the highest prevalence of OE was found in Welsh Springer Spaniels. Compared to the other Spaniels, OE was more often recurrent and more frequently surgically managed in American Cocker Spaniels. Based on the questionnaire, early onset (<1 year) of OE increased the risk of end-stage OE. In American Cocker Spaniels, OE requires an intensive approach from the first treatment, and prevention of recurrence should be emphasised. The causes and treatment of OE in this breed warrant further study.

## Background

Otitis externa (OE) is a relatively common disease in dogs [1]. In previous studies, the estimated prevalence of OE in primary-care veterinary practice has varied from 4.5% [2] and 10.2% [3] in the UK and England to 13% in the US. [4] Successful treatment of OE requires recognition and control of all the causative factors of OE, including primary and secondary causes, as well as predisposing and perpetuating factors [1, 5–7]. Cocker Spaniels have a breed predisposition to OE [8–10]. However, in the literature the term “Cocker Spaniel” is used to describe both English and American Cocker Spaniels; consequently the prevalence of OE in American Cocker Spaniels remains unknown. Some reports suggest, however, that American Cocker Spaniels are overrepresented among breeds that require total ear canal ablation and bulla osteotomy (TECABO) surgery as a treatment of end-stage OE [11–15].

This study aimed to evaluate the prevalence of OE in American Cocker Spaniels in Finland in comparison with other Spaniel breeds. We also aimed to evaluate American Cocker Spaniel owners’ assessments of the clinical signs and management of OE, and to identify potential risk factors for end-stage OE.

## Methods

### Prevalence study

We conducted a retrospective cross-sectional study, reviewing the medical records of all dogs visiting 55 privately owned first opinion small animal veterinary clinics during 2010. The clinics varied in size and geographical location throughout Finland, but shared a database. These clinics covered 17–33% (the percentage varied regionally) of the total companion animal veterinary market in Finland [16]. The total number of dogs in Finland in 2010 is unknown; but according to Statistics Finland, it stood at approximately 630,000 in 2012 [17].

Information retrieved from the database included dog breed, the number of dogs with veterinary consultations (for any reason) and the number of dogs with ear-related consultations (a keyword “ears” as the presenting sign), as well as topical ear-medication prescriptions. Since the keyword “ears” and the use of topical ear medications also included diagnoses other than OE, these figures were only used to determine the relative frequency of ear diseases in different breeds, not the true prevalence. We further classified topical ear medications according to national recommendations from the Finnish Food Safety Authority Evira [18] into first-line (primary) and second-line (secondary) medications. First-line topical ear medications contain miconazole and polymyxin-B or fusidic acid, framycetin and nystatin as antimicrobials. Second-line topical ear medications contain fluoroquinolones or gentamicin and should only be used after bacterial culture and sensitivity testing [18].

In order to determine the prevalence of OE in different Spaniel breeds, we further examined the medical records of American and English Cocker Spaniels as well as Welsh and English Springer Spaniels, and then calculated the number of dogs with at least 1 diagnosed OE episode during 2010. The diagnosis of OE was based on clinical and otoscopic examinations by a veterinarian (this information was extracted from the free text notes), cytology of the aural exudate (when available) and treatment information. The dogs were required to have clinical evidence of OE together with relevant treatment prescription, to be classified as having OE. In most of the cases, the medical record data did not contain sufficient information to rule in or out concurrent otitis media, so we used only one diagnosis term “otitis externa”. In addition, for each breed, we recorded the total number of consultations, the total number of OE episodes in each ear-diseased dog and the number of dogs undergoing ear canal surgery during 2010.

### Owner questionnaire

An online owner questionnaire on ear and skin diseases in American Cocker Spaniels was developed to evaluate the owners´ assessments of the clinical signs and management of OE among this breed. The questionnaire was in Finnish, and a small pilot survey with a subsample of owners evaluated its ease of completion prior to the full-scale launch. Veterinarians, breeders and the American Cocker Spaniel Kennel Club informed private dog owners about the questionnaire from October 2009 to June 2010. Owners were invited to participate in this voluntary and open study regardless of whether their dog had suffered from otitis. The questionnaire was freely downloadable via the Internet and also available in print format. In addition to questions on ear and skin conditions, the questionnaire consisted of multiple-choice questions on signalment (age, sex, colour and weight), overall health and general dog management.

Questions about ear conditions covered the age of onset, clinical signs and the development of OE as noticed by the owner. The owners were also requested whether they had consulted a veterinarian after noticing the signs of otitis. The treatment of OE included questions on possible medication(s), the recurrence of signs after treatment(s) and whether the ears were re-evaluated by a veterinarian following treatment. Table 1 presents the most relevant questions on ears.

**Table 1:** The most relevant questions concerning ears in the American Cocker Spaniel owner questionnaire

Question	Responses								Response rate without I don´t know responses (%)
1. Has your dog suffered from ear or skin problems?	No	Only ear problems	Only skin problems	Both ear and skin problems					100
2. At what age did your dog experience the first signs of otitis?	Less than 3 months	3 months to less than 6 months	6 months to less than 12 months	1 year to less than 3 years	More than 3 years	I don’t know			85
3. What were the most common signs of ear disease?	Scratching of the ears	Redness of the ears (erythema)	Foul smelling ears	Increased ear secretion	Head shaking	Head tilting	Ear soreness	Rash on pinnae	100
4. How many times has your dog been treated for otitis?	Never	Once	Twice	3 times	4–5 times	At least 6 times			100
5. List the medications which you have used to treat your dog´s otitis									91
6. For how long did the signs of otitis subside following treatment?	Less than 1 week	1 week to less than 2 weeks	2 weeks to less than 4 weeks	1–3 months	Signs continued despite treatment	I don’t know			63–75
7. Did you bring your dog for a veterinary re-check after treatment?	Yes, every time	Yes, sometimes after treatment	No						99
8. Has surgery been performed to treat your dog’s ears?	No	Yes, Zepp^a^	Yes, TECABO^a^						87

^a^ *Zepp* ear surgery where only part of the ear canal is removed, *TECABO* ear surgery where the entire ear canal is removed and the bony bulla is opened

Questions about skin diseases covered the age of onset, continuity, seasonality, appearance, localisation and the development of skin lesions as noticed by the owner. The level of pruritus, explained as scratching, biting, chewing, licking and/or rubbing, at the time of answering the questionnaire was solicited using a 0–10 visual analogue scale (modified from Hill et al. [19]).

## Statistical analysis

### Prevalence study

The breeds of dogs were divided into “Spaniel breeds” and “other breeds” as well as into “dogs with pendulous ears” and “dogs with erect ears” according to the appearance of the breed. The group “Spaniel breeds” included all Spaniel breeds in the database. The proportion of ear-related consultations between Spaniels and dogs of other breeds and between dogs with pendulous and erect ears was assessed using univariate logistic regression models. The proportion of first-line and second-line topical ear medication prescriptions were analysed in the same way.

### Owner questionnaire

We defined one dichotomous (yes or no) endpoint for the owner questionnaire database: incidence of end-stage OE. Dogs that were treated with surgery or dogs that were euthanized due to severe OE were classified as having end-stage OE. We determined predefined lists of potential explanatory factors for the response: signalment (age, gender and body weight), diet (commercial diet, raw food diet, veterinary prescription diet for skin patients, use of nutritional oils or use of other additional nutritional supplements), owner’s management of the ears (ear inspection frequency, ear cleaning frequency, frequency of shaving the dog´s pinnae and cheeks, swimming, rapidness of seeking veterinary consultations and whether a veterinary re-evaluation was performed after treatment) and clinical signs of disease (presence of skin lesions, age of onset of first OE episode, ear scratching, ear erythema, foul smell from the ears, increased ear secretion, head shaking, head tilting, rash on the pinnae or ear soreness). Some of these factors had low frequencies in some categories. Therefore, we created a few combinations in order to analyse the data (Table 2).

**Table 2:** Variable modifications for the statistical analysis

Variable	Categories	Action
Age of onset of signs	“Less than 3 months”, “3 months to less than 6 months”, “6 months to less than 12 months”	Combined to “less than 1 year”
Recurrence of signs of otitis	“Less than 1 week”, “1 week to less than 2 weeks”, “2 weeks to less than 4 weeks”	Combined to “less than 4 weeks”
Ear inspection frequency	“Once a week”, “once every 2 weeks”, “more seldom”	Combined to “once a week or more seldom”
Frequency of shaving the dog´s pinnae and cheeks	“Once every fortnight”, “biannually”, “once a year”,”never”	Combined
Rapidness of seeking veterinary advice	“1–3 days after noticing signs of otitis”, “4–7 days after noticing signs of otitis”	Combined
Rapidness of seeking veterinary advice	“8–14 days after noticing signs of otitis”, “I did not seek veterinary advice”	Combined

The data were collected from the American cocker spaniel owner questionnaire

Each explanatory factor was first assessed separately using univariate logistic regression models with the explanatory factor in question as the sole fixed term in the model. Second, a penalised least absolute shrinkage and selection operator (LASSO) logistic regression model [20, 21] was fitted for our response variable. LASSO is a regression analysis method that performs both variable selection and regularisation in order to enhance the predictive accuracy and interpretability of the fitted statistical model. We used Akaike Information Criteria (AIC) for the optimal model selection and the Newton–Raphson Optimisation as the optimisation technique.

Least absolute shrinkage and selection operator regression was applied separately for four different groups of explanatory variables: (1) signalment, (2) diet, (3) owner’s management of the ears and (4) clinical signs.

We calculated the odds ratios (OR) with a 95% confidence interval (CI) to quantify the results. We considered *P* < 0.05 statistically significant. All statistical analyses were completed at 4Pharma Ltd using SAS^®^ System for Windows, version 9.3 (SAS Institute Inc., Cary, NC, USA).

## Results

### Prevalence study

The medical record search yielded information on 98,736 dogs, comprising 220 different breeds and mongrels, which represented 15.7% of the estimated total dog population in Finland [17]. Of these dogs, 11,281 (11.4%) had ear-related consultations. Among the 178 most prevalent breeds in the dataset (with a minimum of 50 individuals receiving veterinary consultations in each breed), the relative frequency of ear-related consultations was highest among Welsh Springer Spaniels (34.2%), followed by Shar-Peis (27.6%) and American Cocker Spaniels (27.1%) (Table 3). The OR for the proportion of dogs having ear-related consultations compared to consultations for other reasons was 1.5 (95% CI 1.4–1.6) in both group comparisons: that is, Spaniel breeds compared to dogs of other breeds and dogs with pendulous ears compared to dogs with erect ears.

**Table 3:** Relative frequency of ear-related consultations and topical ear medication prescriptions in different dog breeds

Breed	Dogs with veterinary consultations	Ear-related consultations n % (95% CI)		Topical ear medication prescriptions n % (95% CI)		Second-line^a^ topical ear medication prescriptions n % (95% CI)	
Welsh Springer Spaniel	468	160	34.2 (29.9–38.5)	136	29.1 (25.0–33.2)	29	6.2 (4.0–8.4)
Shar-Pei	76	21	27.6 (17.6–37.7)	21	27.6 (17.6–37.7)	8	10.5 (3.6–17.4)
American Cocker Spaniel	329	89	27.1 (22.3–31.9)	75	22.8 (18.3–27.3)	29	8.8 (5.7–11.9)
West Highland White Terrier	1341	361	26.9 (24.5–29.3)	314	23.4 (21.1–25.7)	63	4.7 (3.6–4.8)
Bullmastiff	243	60	24.7 (19.3–30.1)	46	18.9 (14.0–23.8)	14	5.8 (2.9–8.7)
Pug	763	187	24.5 (21.4–27.6)	156	20.4 (17.5–23.3)	30	3.9 (2.5–5.3)
Dogue de Bordeaux	98	24	24.5 (16.0–33.0)	18	18.4 (10.7–26.1)	2	2.0 (−0.8 to 4.8)
Basset Hound	147	34	23.1 (16.3–29.9)	29	19.7 (13.3–26.1)	8	5.4 (1.7–9.1)
Grand Basset Griffon Vendeen	59	13	22.0 (11.4–32.6)	13	22.0 (11.4–32.5)	2	3.4 (−1.2 to 8.0)
Dogo Argentino	97	21	21.6 (13.4–29.8)	23	23.7 (15.2–32.2)	2	2.1 (−0.8 to 5.0)
Bull Terrier	188	40	21.3 (15.4–27.2)	33	17.6 (12.2–23.0)	11	5.9 (2.5–9.3)
Bulldog	596	122	20.5 (17.3–23.7)	105	17.6 (14.5–20.7)	27	4.5 (2.8–6.2)
Labrador Retriever	3708	737	19.9 (18.6–21.2)	644	17.4 (16.2–18.6)	75	2.0 (1.5–2.5)
Lagotto Romagnolo	426	84	19.7 (15.9–23.5)	70	16.4 (12.9–19.9)	3	0.7 (−0.1 to 1.5)
English Springer Spaniel	491	97	19.8 (16.3–23.3)	89	18.1 (14.7–21.5)	28	5.7 (3.6–7.5)
English Cocker Spaniel	1467	274	18.7 (16.7–20.7)	165	15.1 (13.3–16.9)	56	3.8 (2.8–4.8)
Swedish Elkhound	289	8	2.8 (0.9–4.7)	5	1.7 (0.2–3.2)	0	0
Japanese Spitz	344	9	2.6 (0.9–4.3)	12	3.5 (1.6–5.4)	2	0.6 (−0.2 to 1.4)
Greyhound	187	4	2.1 (0.0–4.2)	2	1.1 (−0.4 to 2.6)	0	0
German Spitz (klein)	171	3	1.8 (−0.2 to 3.8)	3	1.8 (−0.2 to 3.8)	0	0
Norrbottenspets	140	2	1.4 (−0.5 to 3.3)	3	2.1 (−0.3 to 4.5)	1	0.7 (−0.7 to 2.1)
All dogs (220 breeds)	98,736	11,281	11.4 (11.2–11.6)	8761	8.9 (8.7–9.1)	1136	1.2 (1.1–1.3)

This table presents the relative frequencies for all dogs and for the 16 most prevalent and 5 least prevalent breeds with ear-related consultations for the year 2010, from the medical records of 55 private veterinary clinics in Finland. n = the number of dogs

^a^A topical product containing marbofloxacin, clotrimazole and dexamethasone (Aurizon^®^, Vetoquinol S.A., Cedex, France); gentamicin, betamethasone and clotrimazole (Otomax^®^, Intervet International B.V., Boxmeer, the Netherlands); or hydrocortisone aceponate, gentamicin and miconazole (Easotic^®^, Virbac, Carros, France)

Of the 98,736 dogs, 8761 (8.9%) were treated with topical ear therapy. First-line topical ear medications were prescribed to 8041 (8.1%) dogs and second-line ear-medications to 1136 (1.2%) dogs, while 416 dogs (0.4%) received both (Table 3). Second-line topical ear medications were prescribed at a higher proportion to Shar-Peis (10.5%) and American Cocker Spaniels (8.8%). When Spaniel breeds were compared to other breeds, the OR of first-line topical ear medications was 1.4 (95% CI 1.3–1.6), while second-line topical ear medications resulted in an OR of 2.6 (95% CI 2.2–3.0). Comparing dogs with pendulous and erect ears, the ORs of first and second-line topical ear medications were 1.7 (95% CI 1.6–1.8) and 1.6 (95% CI 1.4–1.9), respectively.

In Cocker and Springer Spaniel breeds, the prevalence of OE was highest in Welsh Springer Spaniels, but the mean number of OE episodes was 1.5-fold higher in ear-diseased American Cocker Spaniels compared with Welsh Springer Spaniels (Table 4). Ear surgery was performed on eight of 89 (9.0%) American Cocker Spaniels, consisting of a Zepp operation (n = 2), a vertical ear canal ablation (n = 1) or TECABO (n = 5). TECABO (n = 1) or Zepp (n = 1) was also performed on two of 231 (0.9%) English Cocker Spaniels, while one of 96 (1.0%) English Springer Spaniels underwent the Zepp operation. None of the Welsh Springer Spaniels underwent surgery.

**Table 4:** The prevalence of otitis externa (OE) in Cocker and Springer Spaniels in Finland

Breed	Number of dogs with veterinary consultations	Number of dogs with diagnosed OE	Prevalence of OE (95% CI)	Total number of veterinary consultations	Total number of OE episodes	Mean number of OE episodes in ear-diseased dogs	Median number of OE episodes in ear-diseased dogs
American Cocker Spaniel	329	89	27.0 (22.2–31.7)	847	231	2.6	2.0
Welsh Springer Spaniel	468	149	31.8 (27.6–36.0)	1286	257	1.7	1.0
English Springer Spaniel	491	96	19.6 (16.1–23.1)	1176	196	2.0	1.0
English Cocker Spaniel	1467	231	15.7 (13.8–17.6)	3609	449	1.9	1.0

The data were collected from the medical records of 55 private veterinary clinics in Finland during 2010

### Owner questionnaire

Questionnaires were received from 117 owners, providing information on 151 dogs. This comprises approximately 10% of the American Cocker Spaniel population in Finland (American Cocker Spaniel Kennel Club, personal communication). Among these, 67 (44%) dogs were male and 84 (56%) were female. Their ages ranged from 0.3 to 14.4 years (mean 4.9 years, SD ± 3.15). In total, 25 of the dogs had already been euthanized, five due to serious ear disease. Of the 151 dogs, 85 (56%) had suffered from OE and 51 (34%) from skin disease. Only 6 dogs (4%) had experienced skin lesions without OE.

### Clinical signs of ear and skin disease

Approximately half of the dogs with OE (47%, 40 out of 85) had no concurrent reported skin lesions. Most dogs (88%, 75 out of 85) had bilateral ear disease. The first episode of OE appeared before the age of 1 year in 46% (33 out of 72) of the dogs, 39% (13 out of 33) of these dogs also showed skin lesions before the age of 1 year and 12% (4 out of 33) after the age of 1 year. Only 22% (16 out of 72) of the dogs displayed the first signs of OE after the age of 3 years.

Half of the ear-diseased dogs’ owners (54%, 46 out of 85) examined their dog’s ears 1–2 times per week and 37% (31 out of 85) every day. The most common reported sign of OE was scratching of the ears (85%, 72 out of 85) followed by a foul smell (62%, 53 out of 75). Of the symptoms related to the skin, scratching (63%, 32 out of 51) and scaling (33%, 17 out of 51) were most commonly reported. The most frequent locations of the skin lesions were the axillae (29%, 15 out of 51), the back (25%, 13 out of 51), the interdigital skin (22%, 11 out of 51) and the paws (20%, 10 out of 51). The pruritus score ranged from 1 to 10, but pruritus was mild or moderate in most of the dogs (mean 4.8, SD ± 2.59).

### Treatment of the ears

After noticing signs of otitis, 94% of the owners (80 out of 85) consulted a veterinarian. Altogether, topical ear medications were used in 79 (93%) dogs with OE, of these 27 (32%) received second-line medications. Half of the ear-diseased dogs (49%, 42 out of 85) received systemic antibiotics, most often amoxicillin and clavulanic acid or cephalexin. Systemic glucocorticoids were used on 19 (22%) and cyclosporine on three (4%) of 85 dogs. Ear cleaning under sedation or anaesthesia was performed on 22 (26%) of 85 dogs.

Most ear-diseased dogs´ owners (81%, 65 out of 80) received instructions from their veterinarian regarding how to clean their dog’s ears at home. Almost all owners (84%, 71 out of 85) used commercially available cleaning products to clean their dog’s ears. The most common product mentioned (81%, 57 out of 70) contained salicylic acid and EDTA (Epi-Otic^®^, Virbac, Carros, France).

### Recurrence of signs

A veterinarian re-evaluated the ears each time after treatment for 32% (25 out of 79) and occasionally among 39% (31 out of 79) of the dogs; but, 29% (23 out of 79) of the owners never brought their dog for a re-evaluation. Approximately half of dog owners (48%, 41 out of 85) reported that the signs of OE resolved for a period of at least 1–3 months after treatment. Signs of OE recurred rapidly within less than 4 weeks or continued despite any medical therapy in 20 of 85 dogs (24%). The incidence of medically unmanageable end-stage OE was 16% (14 out of 85). Surgery was performed on 11 (13%) dogs consisting of either Zepp (n = 5) or TECABO (n = 6). Two dogs undergoing Zepp and three other dogs were euthanized due to severe OE.

### Diet and general health

A commercial pet diet was fed to 90% (136 out of 151) of the dogs, either exclusively or in conjunction with home-cooked food. In addition, 8% of the dogs (12 out of 151) received only home-cooked or raw food. Among those who were on a commercial diet, a veterinary prescription diet formulated for skin patients was fed to 13 dogs–nine dogs with both ear and skin symptoms, three dogs with only skin symptoms and one dog with only OE. Nearly one-quarter of the dogs (23%, 35 out of 151) received nutritional oils and 11% (16 out of 151) received other nutritional supplements. One-third of the dogs (31%, 46 out of 151) had other illnesses such as hypothyroidism, cardiac failure, epilepsy, orthopaedic conditions and ophthalmic disease. Furthermore, eight of 151 (5%) dogs suffered from hypothyroidism, seven of which had both ear and skin symptoms, although none of these had end-stage OE.

### Factors associated with end-stage OE

**Fig. 1:**
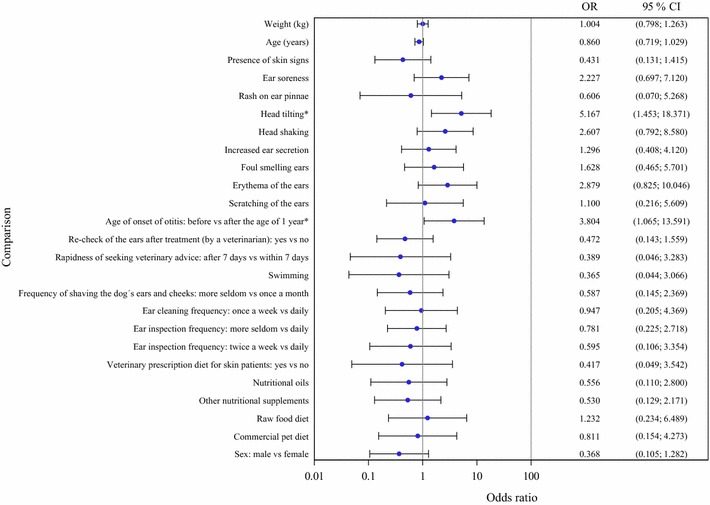
Explanatory factors for end-stage otitis in the univariate logistic regression analyses. The statistically significant factors are marked with* asterisk*

Based on the univariate logistic regression analyses, the onset OE before the age of 1 year (*P* = 0.040, OR = 3.804) and head tilting (*P* = 0.011, OR = 5.167) were risk factors for end-stage OE (Fig. 1). In the multivariate logistic regression model, in addition to head tilting (OR = 7.111) and onset of OE before the age of 1 year (OR = 2.326), erythema of the ears (OR = 1.460) and ear soreness (OR = 1.226) increased the risk, but the presence of symptoms related to the skin decreased (OR = 0.641) the risk of end-stage OE. None of the variables in the signalment, diet and owner’s management of the ears groups were identified as risk factors for end-stage OE in the logistic regression analyses.

## Discussion

In this study, the prevalence of otitis externa among American Cocker Spaniels visiting 55 private veterinary clinics in Finland during 2010 was high, reaching 27%. This is 1.4- to 5.9-fold higher than the figures reported in other studies for Cocker Spaniels [3, 22], for dogs with pendulous hairy ears [23] or for dogs in general [2–4]. Furthermore, among all breeds of dogs, ear-related consultations were most frequent in Welsh Springer Spaniels, followed by Shar-Peis and American Cocker Spaniels. Our finding that Spaniel breeds, compared to other dog breeds, had an increased risk for ear-related consultations and for the use of topical ear medications is in line with previous studies showing a predisposition to OE among these breeds [8–10].

When the four Spaniel breeds were studied more closely, American Cocker Spaniels and Welsh Springer Spaniels suffered more often from OE than English Cocker and Springer Spaniels. Interestingly, the highest prevalence of OE occurred among Welsh Springer Spaniels. To our knowledge, the high prevalence of OE in this breed has not been documented previously, a finding that warrants further study. However, our study reflected the situation during 1 year only and the 95% confidence intervals of the prevalence estimates of American Cocker Spaniels overlapped with those of English and Welsh Springer Spaniels. Thus, the role of chance in sampling should be remembered within these results. The mean and median number of OE episodes in ear-diseased dogs were highest among American Cocker Spaniels, which may indicate that OE recurs more often in this breed. Moreover, the number of ear surgeries on American Cocker Spaniels was high in comparison to other Spaniels, indicating that OE was also more difficult to manage medically in this breed. Difficulties in treatment were also reported by owners, whereby 24% of the owners of the ear-diseased dogs reported that the signs of OE recurred within one month or continued despite treatment. Furthermore, in 16% of the dogs, medical treatment failed, and 5.6% of dogs were euthanized due to severe OE.

According to the owner questionnaire, half of the dogs with OE displayed initial signs of OE before reaching 1 year of age. This could be explained in part by predisposing anatomical factors. Cocker Spaniels have long, hairy pendulous pinnae and, compared to Greyhounds and mongrels, a greater density of compound hair follicles and ceruminous glands in the ear canal [24]. Pendulous conformation is considered a predisposing factor for OE [25, 26], as also found in our study. In addition, the accumulation of cerumen in the external ear canal is thought to predispose dogs to OE [5], and sometimes the only underlying cause for chronic OE appears to be excessive cerumen production [1].

The early onset of first OE before the age of 1 year was a significant risk factor for end-stage OE. This is an interesting finding and, to our knowledge, not previously reported. It is possible that genetic predisposing factors play a larger role in the development of end-stage OE in this breed than previously understood. Other risk factors identified for end-stage OE such as ear soreness and head tilting appear to be clinical signs rather than risk factors for chronic otitis. These signs merely indicate that such dogs most probably suffered from severe otitis externa and otitis media. It is known that on-going inflammation can lead to permanent changes in the physiology and microanatomy of the ear canal [6]. American Cocker Spaniels in particular have a strong breed predisposition to proliferative ceruminous gland hyperplasia and ectasia, resulting in end-stage OE without proper care [13]. These facts and the results of this study imply that, in order to reduce the likelihood of chronic changes, the treatment of OE in a young American Cocker Spaniel should be intensive from the very first treatment, with greater emphasis placed on preventing recurrence. Unfortunately, after ear treatment many dogs did not undergo a re-evaluation. It is uncertain, however, whether a re-evaluation was requested. The re-check of the ears is of utmost importance in recognising and controlling perpetuating factors [27]. Increasing the frequency of veterinary re-checks through client and veterinary education might lead to better outcomes.

Skin symptoms were reported in only half of the dogs with OE; 29% of these dogs showed initial signs of OE before the age of 1 year. Interestingly, the presence of symptoms related to the skin decreased the risk of end-stage OE. Possible explanations for this surprising finding are that veterinarians may have neglected determining the primary cause of OE in dogs without concurrent skin disease, or that the primary cause of OE may have been more difficult to identify or control in these dogs. Thus, it would be useful to identify the primary cause of OE, particularly in these cases, through a more detailed study. In 1994, Rosychuck described “idiopathic inflammatory or hyperplastic otitis” in American Cocker Spaniels with chronic OE without other significant skin disease, but the cause of this ear disease remains unknown [28]. In our study, veterinary prescription diets for skin patients were fed more often to dogs with concurrent skin disease, suggesting that hypersensitivities were not considered a likely primary cause in dogs with OE only. However, allergies have been noted as the most common primary cause of OE [9, 10] and Cocker Spaniels are predisposed to atopic dermatitis [29]. Furthermore, food allergies [30] and canine atopic dermatitis [31] may be manifested solely by OE or otitis may precede other signs in allergic dermatitis [32]. An undiagnosed allergic disease may have been the primary cause of recurrent OE in some of the dogs in our study. Our results underline the need for a thorough investigation to identify the primary cause of otitis in all cases of recurrent OE.

We should consider the results in relation to limitations of our study. In the prevalence study, the diagnosis of OE was based on medical record data. Thus, it was not possible to verify the accuracy of the diagnosis. Because we distributed the owner questionnaire through veterinarians, breeders and the American Cocker Spaniel Kennel Club, we may not have reached all owners. The differences in the prevalence estimates of OE among American Cocker Spaniels between the questionnaire and the prevalence study indicate that the owners of dogs with ear-disease may have been more willing to participate in the questionnaire study, resulting in a nonresponse error. In addition, responses may have been influenced by the owners’ perceptions or memory of various facts. However, the prevalence estimates of ear-diseased dogs receiving second-line topical ear medications and undergoing surgery were similar in both studies. The temporal sequence of events can be difficult to establish in cross sectional studies. Also in our study, there was potential reverse causality for some of the factors, such as ear-cleaning, because it was not known, whether ear-cleaning preceded OE or was introduced as a treatment of OE. In addition, certain factors, such as owner’s management of the ears, may have changed over time, but data were only captured for one point of time. Some questions appeared to be more difficult to answer, resulting in a lower response rate and a higher number of “I don’t know” responses, thus increasing measurement error. The problem regarding missing data was greater in the multivariate analyses, since the analysis can only be conducted using complete data for all of the explanatory variables included in our model. Reducing the number of observations rendered the results less reliable and, similarly, the results between the univariate and multivariate analyses were not completely comparable since they relied on somewhat different data. Because our questionnaire was freely downloadable from the internet, the total survey error is impossible to calculate. These limitations, many of which are inherent for cross sectional studies, should be kept in mind when interpreting our results.

## Conclusions

We conclude that the prevalence of OE in American Cocker Spaniels in Finland was higher than previously reported among Cocker Spaniels. Interestingly, the highest prevalence of OE was found in Welsh Springer Spaniels. The recurrence of OE and the number of ear surgeries were highest among American Cocker Spaniels in comparison with other spaniels. Based on an owner questionnaire, half of the American Cocker Spaniels displayed the initial signs of OE before the age of 1 year and half of the dogs with OE showed no concurrent skin lesions. Furthermore, the onset of OE before the age of 1 year significantly increased the risk of end-stage OE. In this breed, OE requires an intensive approach from the first treatment, and emphasis should be placed on preventing recurrence. Further studies should focus on the causes and treatment of OE in this breed.
